# Identification of potential LncRNAs as papillary thyroid carcinoma biomarkers based on integrated bioinformatics analysis using TCGA and RNA sequencing data

**DOI:** 10.1038/s41598-023-30086-0

**Published:** 2023-03-16

**Authors:** Jia-Lin Feng, Wen-Jie Zheng, Le Xu, Qin-Yi Zhou, Jun Chen

**Affiliations:** Department of Head and Neck Surgery, Ren Ji Hospital, Shanghai Jiao Tong University School of Medicine, Shanghai, China

**Keywords:** Cancer, Computational biology and bioinformatics

## Abstract

The roles and mechanisms of long non-coding RNAs (lncRNAs) in papillary thyroid cancer (PTC) remain elusive. We obtained RNA sequencing (RNA-seq) data of surgical PTC specimens from patients with thyroid cancer (THCA; n = 20) and identified differentially expressed genes (DEGs) between cancer and cancer-adjacent tissue samples. We identified 2309 DEGs (1372 significantly upregulated and 937 significantly downregulated). We performed Gene Ontology, Kyoto Encyclopedia of Genes and Genomes, gene set enrichment, and protein–protein interaction network analyses and screened for hub lncRNAs. Using the same methods, we analyzed the RNA-seq data from THCA dataset in The Cancer Genome Atlas (TCGA) database to identify differentially expressed lncRNAs. We identified 15 key differentially expressed lncRNAs and pathways that were closely related to PTC. Subsequently, by intersecting the differentially expressed lncRNAs with hub lncRNAs, we identified LINC02407 as the key lncRNA. Assessment of the associated clinical characteristics and prognostic correlations revealed a close correlation between LINC02407 expression and N stage of patients. Furthermore, receiver operating characteristic curve analysis showed that LINC02407 could better distinguish between cancerous and cancer-adjacent tissues in THCA patients. In conclusion, our findings suggest that LINC02407 is a potential biomarker for PTC diagnosis and the prediction of lymph node metastasis.

## Introduction

Thyroid cancer (THCA) is the most common malignant tumor of the endocrine system, with morbidity and mortality accounting for approximately 95% and 67%, respectively, of all endocrine tumors. Worldwide, nearly 570,000 patients are diagnosed with THCA annually^1,2^. Clinically, papillary carcinoma is the most common type of THCA, accounting for over 85% of all THCAs^3^. Although papillary thyroid cancer (PTC) shows a low malignancy and good prognosis, approximately 25% of patients with PTC experience post-surgery recurrence during long-term follow-up beyond 20 years, which is associated with increased mortality^4,5^. In addition, some patients with locally advanced PTC show invasion of the surrounding tissues, distant metastasis, and resistance to radioactive iodine therapy, with a 10-year survival rate of < 10%^6^. Therefore, exploring the mechanisms underlying PTC occurrence and devising therapeutic targets for PTC are important for improving the efficacy of therapeutic strategies against PTC and prolonging patient survival.

Several studies have demonstrated the potential of long non-coding RNAs (lncRNAs), which are non-coding transcripts more than 200 nucleotides long, in cancer diagnosis and therapy. They participate in several signaling pathways and are involved in the regulation of various cellular functions, such as apoptosis, cell cycle progression, proliferation, migration, and invasion, through epigenetic, transcriptional, and post-transcriptional regulation^7–10^. Chen et al.^11^ also introduced that lncRNAs can participate in almost the entire life cycle of cells through different mechanisms. Mutation and dysregulation of lncRNAs can lead to the development of various complex human diseases. Owing to their functional diversity and specific expression in cancer tissues, they have received increased interest in cancer biomarker research.

Several lncRNAs associated with the development of THCA, particularly PTC, have been identified. For instance, linc00941 controls CDH6 activity in PTCs and inhibits autophagy by promoting the cytoskeletal rearrangements required for invasiveness^12^. Feng et al.^13^ have demonstrated the potential of lncRNA n384546 that promotes PTC progression and metastasis by acting as a competing endogenous RNA (ceRNA) via the miR-145-5p/AKT3 axis as a therapeutic target in patients with PTC. Similarly, lncRNA AB074169 acts as a tumor suppressor that impairs PTC cell proliferation by inhibiting DNA replication and regulating the expression of cell cycle-related genes^14^. Goedert et al.^15^ used data from patients with THCA (deposited in The Cancer Genome Atlas [TCGA] database) to identify differentially expressed lncRNAs related to the BRAF V600E mutation through bioinformatic analysis and found that the targets of BRAF V600E-related lncRNAs were mainly involved in the calcium signaling pathway, extracellular matrix–receptor interactions, and the mitogen-activated protein kinase (MAPK) pathway. Xu et al.^16^ performed Gene Ontology (GO) and Kyoto Encyclopedia of Genes and Genomes (KEGG) analyses of thyroid-tissue RNA profiles in TCGA database. They constructed a competing endogenous RNA (ceRNA) network of mRNAs, lncRNAs, and microRNAs (miRNAs) using miRDB, miRTarBase, and TargetScan databases. This study identified two lncRNAs (MIR1281A2HG and OPCML-IT1) that were significantly associated with overall survival (OS) in patients with THCA.

However, previous studies have only used molecular biology methods or bioinformatics analysis methods, providing limited knowledge. Integrated analyses using different tools and different tissue samples could provide robust results. In this study, we aimed to discover key lncRNAs to aid in the early diagnosis of PTC and the discovery of potential therapeutic targets by analyzing the RNA sequencing data of surgical specimens of patients with THCA and the THCA dataset in TCGA database combined with downstream analyses using several bioinformatic tools. First, we performed high-throughput sequencing of surgical PTC specimens to obtain the primary data. We then performed differential gene expression analysis and screened for hub lncRNAs through GO, KEGG, gene set enrichment analysis (GSEA), and protein–protein interaction (PPI) network analyses. Second, we analyzed data from patients with THCA (from TCGA database) using the above-mentioned methods to identify differentially expressed lncRNAs. Finally, we intersected the differentially expressed lncRNAs with hub lncRNAs to identify the key lncRNAs and analyzed their clinical characteristics and prognostic value.

## Results

### Differential expression analysis based on RNA sequencing (RNA-Seq) data of surgical specimens obtained from patients with THCA

**Figure 1:**
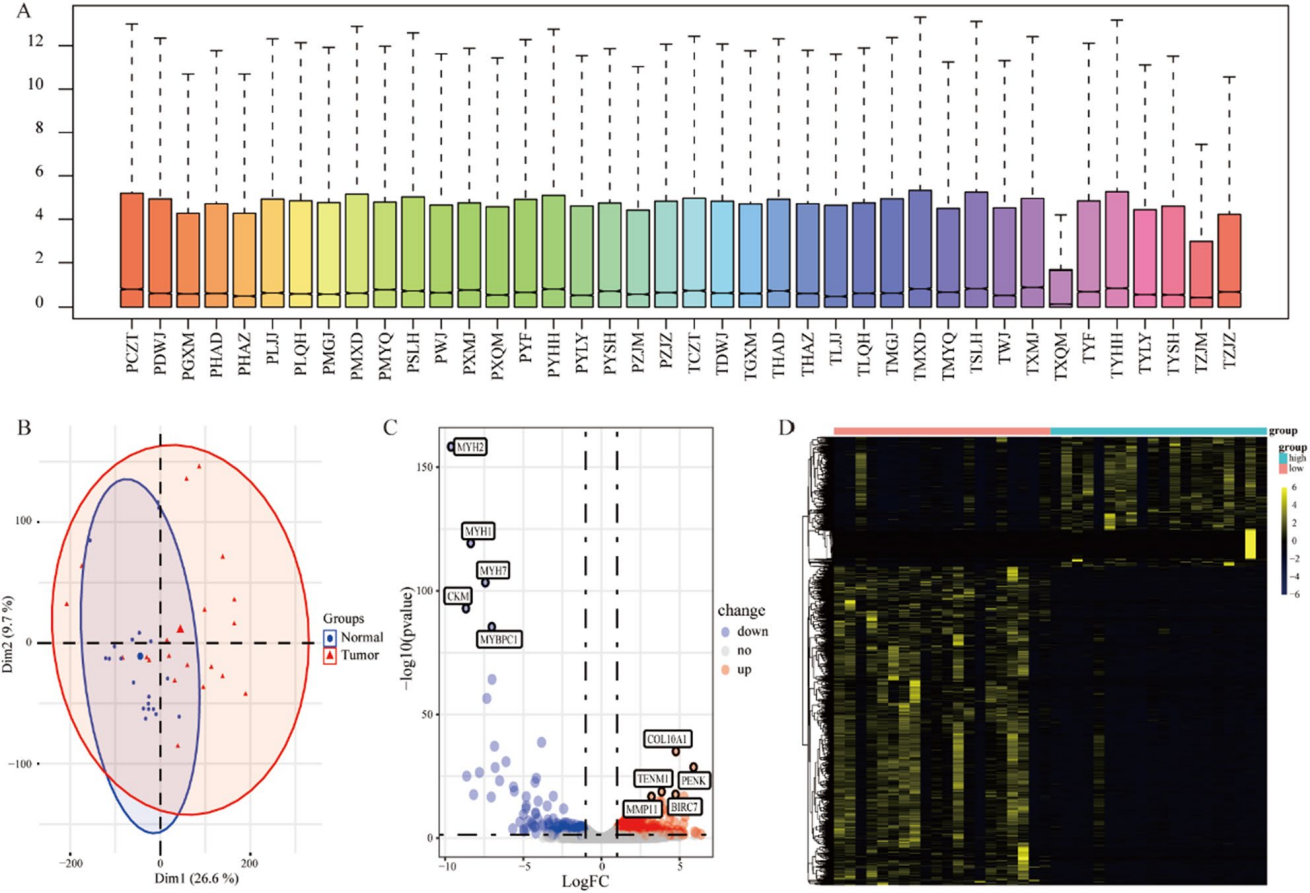
Differential gene expression analysis based on RNA sequencing (RNA-Seq) data of specimens collected from patients with THCA. (**A**) Boxplots show the comprehensive range of FPKM values for different genes in each tissue. (**B**) Principal component analysis (PCA) plots show the differences in gene expression in cancer and cancer-adjacent tissue samples. (**C**) Volcano plots and (**D**) heatmaps showing the respective expression levels of differentially expressed genes (DEGs) between cancer and cancer-adjacent tissues of patients with THCA.

We performed RNA sequencing (RNA-Seq) analysis of 20 pairs of cancer and cancer-adjacent tissue samples obtained from THCA patients. The boxplots show the distribution of fragments per kilobase of transcript per million mapped reads (FPKM) for each sample (Fig. 1A). Principal component analysis (PCA) revealed differences between cancerous and cancer-adjacent tissues in patients with THCA (Fig. 1B). Subsequently, we identified differentially expressed genes (DEGs) in cancer and cancer-adjacent tissues. Screening with the criteria of |log_2_ fold-change (log_2_ FC)||≥ 1 and q < 0.05 yielded 2309 DEGs, of which 1372 were significantly upregulated and 937 were significantly downregulated in the THCA patient tissues. The volcano plots and heat maps are shown in Fig. 1C and D, respectively.

### Prediction and functional enrichment analysis of differential lncRNA target genes

To identify differentially expressed lncRNA target genes, we performed cis- and trans-target analyses to indirectly predict their functions. Cis-target gene prediction is mainly based on the correlation between the function of an lncRNA and its adjacent protein-coding genes at the genomic locus. Trans-target gene prediction is primarily based on the fact that the function of an lncRNA does not depend on its positional relationship with the coding gene, but rather on its correlation with the co-expressed gene. Finally, we identified target genes corresponding to the differentially expressed lncRNAs.

**Figure 2:**
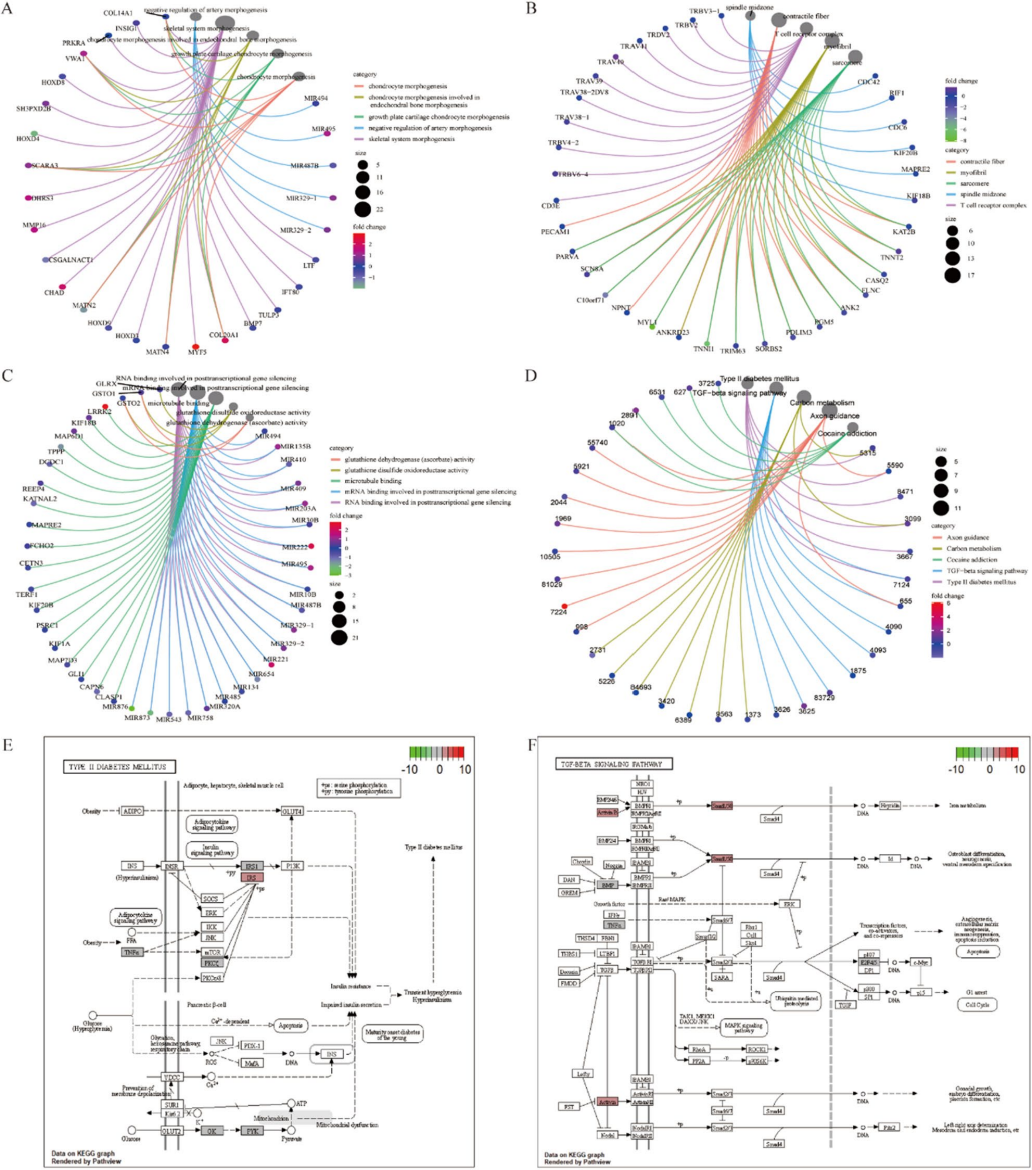
Functional enrichment analysis of cis-target genes based on differential lncRNAs. (**A**–**C**) Based on the three GO classifications (CC, BP, and MF), our analysis suggests that the expression of cis-target genes is related to several terms, such as negative regulation of artery morphogenesis, spindle midzone, RNA binding involved in post-transcriptional gene silencing, and other biologically related processes. (**D**) KEGG enrichment analysis indicates that the expression of cis-target genes is significantly associated with different pathway terms, such as type II diabetes mellitus, the TGF-beta signaling pathway, and other pathways. (**E**,** F**) Visual display of changes in the relevant enriched pathways.

Subsequently, we performed a functional enrichment analysis of the cis- and trans-target genes of the differentially expressed lncRNAs. GO analysis based on biological processes (BPs), molecular functions (MFs), and cellular components (CCs) pathways showed that cis-target genes were closely related to BP terms, such as negative regulation of artery morphogenesis, spindle mid zone, and RNA binding involved in post-transcriptional gene silencing (Fig. 2A–C). The results of KEGG functional analysis suggested that the expression of these target genes mainly affected type II diabetes mellitus, the transforming growth factor-beta (TGF-β) signaling pathway, and other pathways (Fig. 2D). The specific path conditions are shown in Fig. 2E and F, respectively.

**Figure 3:**
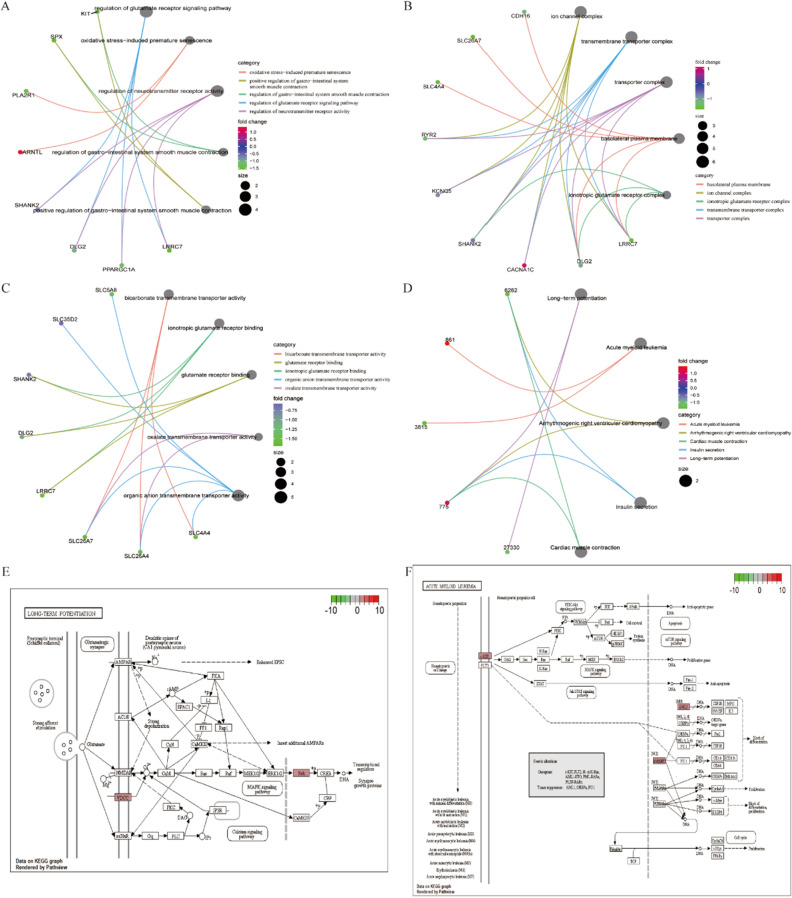
Functional enrichment analysis of trans-target genes based on differentially expressed lncRNAs. (**A**–**C**) Based on the three GO classifications (CC, BP, and MF), our analysis suggests that the expression of trans-target genes is associated with the regulation of glutamate receptor signaling pathway, ion channel complex, and bicarbonate transmembrane transporter activity BP terms. (**D**) KEGG enrichment analysis shows that the expression of trans-target genes is significantly associated with different pathway terms, such as long-term potentiation, acute myeloid leukemia, and other pathways. (**E**, **F**) Visual display of changes in the relevant enriched pathways.

Functional enrichment analysis of trans-target genes based on the BP, CC, and MF pathways identified their association with BP terms, including regulation of the glutamate receptor signaling pathway, ion channel complex, and bicarbonate transmembrane transporter activity (Fig. 3A–C). KEGG functional analysis suggested that the expression of these target genes mainly affected long-term potentiation, acute myeloid leukemia, and other pathways (Fig. 3D). Specific pathways are shown in Fig. 3E and F.

**Figure 4:**
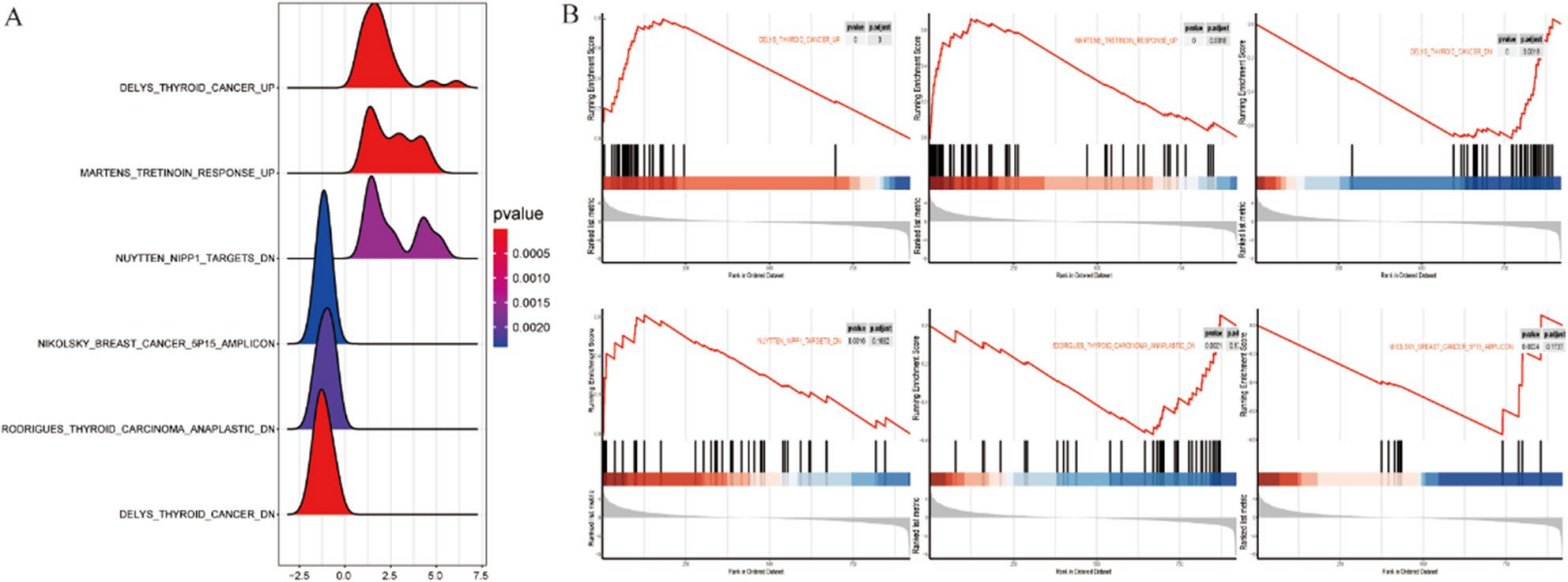
GSEA of all target genes based on differential lncRNAs. (**A**) Volcano map showing an overview of the GSEA results. (**B**) The results show that pathways (such as delys thyroid cancer up, martens tretinoin response up, and nuytten NPP1 targets dn) were significantly enriched in tumor tissues from patients with THCA. In contrast, the delys thyroid cancer dn, Rodrigues thyroid carcinoma anaplastic dn, and Nikolsky breast cancer 5p15 amplicon pathways were significantly downregulated in tumor tissues from patients with THCA.

Simultaneously, we performed gene set-enrichment analysis (GSEA) to study the expression of all target genes in cancer and cancer-adjacent tissues. GSEA showed that THCA up, martens tretinoin response up, nuytten NPP1 targets dn, and other GSEA pathways were significantly enriched, and the THCA dn, thyroid carcinoma anaplastic dn, and Nikolsky breast cancer 5p15 amplicon pathways were significantly downregulated in the tumor tissue of patients with THCA (Fig. 4A). A specific pathway diagram is shown in Fig. 4B.

### Construction of ceRNA regulatory network and protein–protein interaction (PPI) network map and hub-gene screening

**Figure 5:**
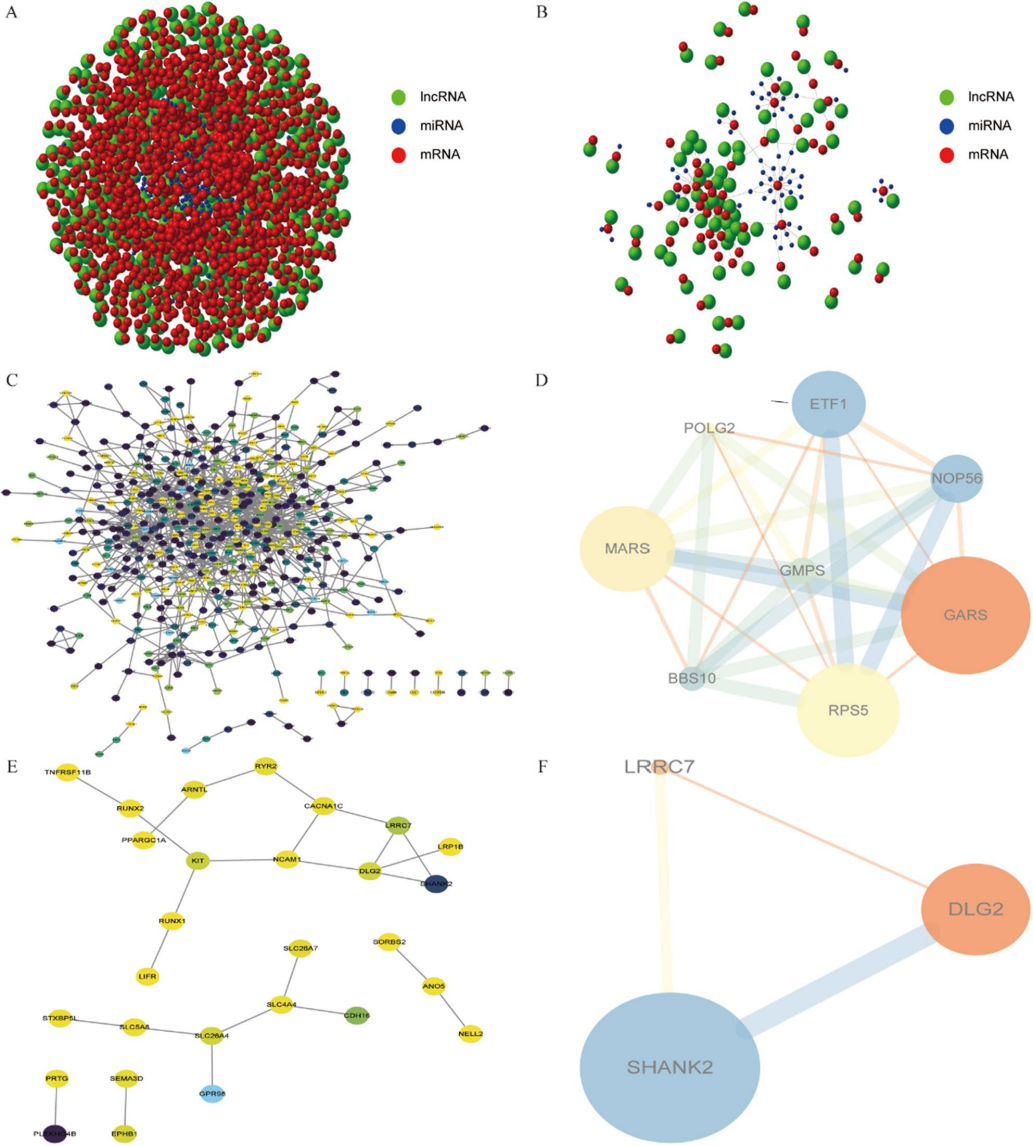
Construction of a ceRNA regulatory network and PPI network. (**A**, **B**) ceRNA networks for the mutual regulation of lncRNA-mRNA-miRNA interactions based on the cis- and trans-target genes of the differential lncRNAs; Green, lncRNAs; Blue, miRNAs; Red, mRNAs. (**C**) PPI network of cis-target genes analyzed using the STRING database, where each node represents a different gene. (**D**) Local high-density regions identified from the PPI network using the MCODE algorithm and used as hub genes. (**E**) PPI network of the trans-target genes using the STRING database, where each node represents a different gene. (**F**) Local high-density regions identified from the PPI network using the MCODE algorithm and used as hub genes, where the sizes of the circles and lines are proportional to the log_2_ FC. The degree of shading was proportional to the *P* value.

**Figure 6:**
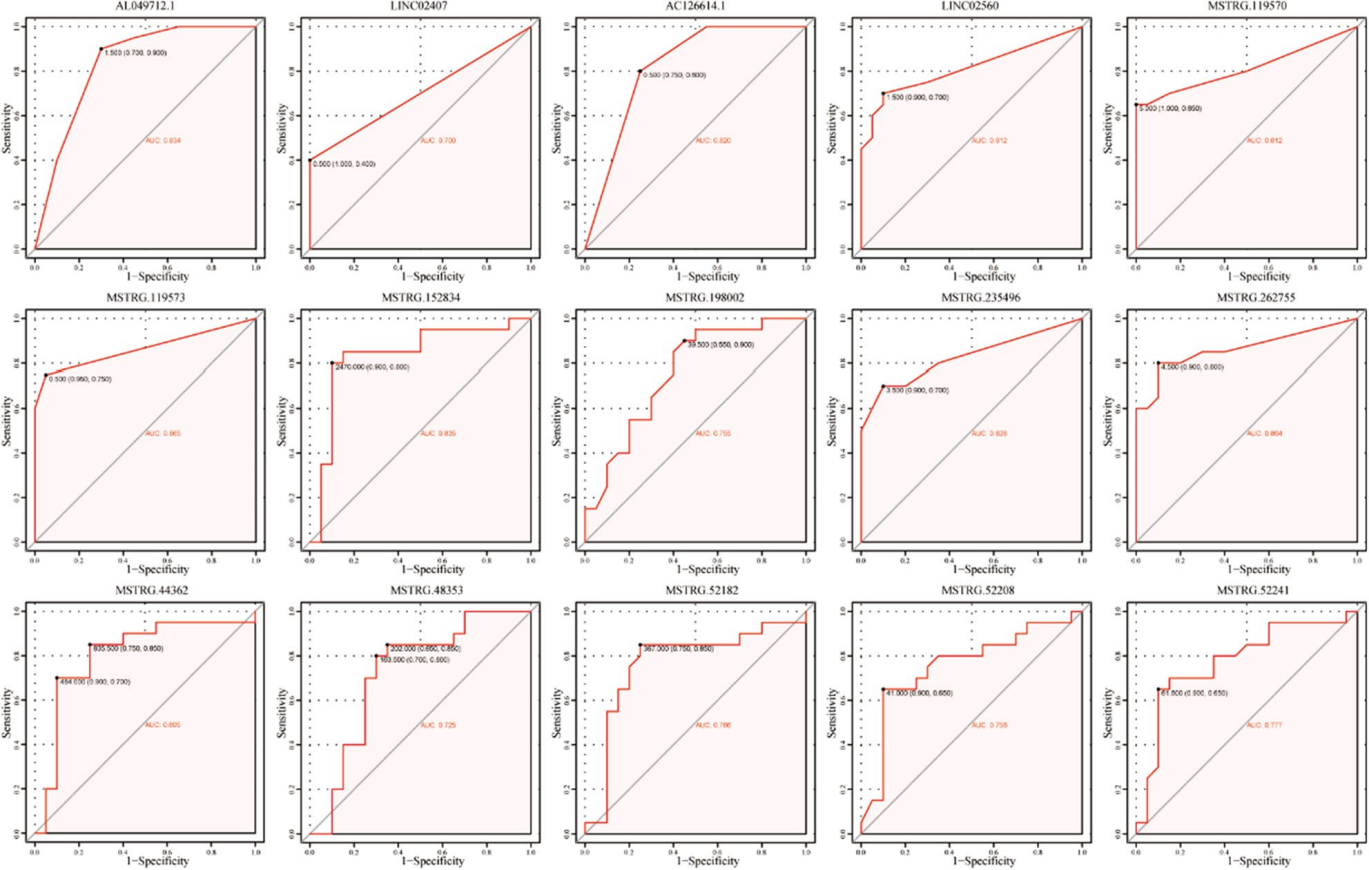
ROC curve analysis of key lncRNAs (n = 15) identified based on hub genes. ROC curve analysis of the 15 key lncRNAs shows good discrimination between cancer and cancer-adjacent tissues from patients with THCA.

We constructed a ceRNA network to understand the mutual regulation of cis- and trans-target genes based on differentially expressed lncRNAs and their corresponding target gene mRNAs combined with the mutually regulated miRNAs predicted by the miRTarBase database^17^ (https://mirtarbase.cuhk.edu.cn/) (Fig. 5A and B). Subsequently, we inputted the cis- and trans-target genes into the Search Tool for Retrieving Interacting Genes (STRING) database^18^ (https://string-db.org) to establish a PPI network between eigengenes (Fig. 5C and E) and selected local dense regions from the PPI network using Cytoscape’s MCODE plugin as the hub genes of the PPI network (Fig. 5D and F). Based on the hub genes obtained by Cytoscape analysis and the regulatory relationship between lncRNAs and target genes, we identified 15 key differentially expressed lncRNAs: AL049712.1, LINC02407, AC126614.1, LINC02560, MSTRG.119570, MSTRG.119573, MSTRG.152834, MSTRG.198002, MSTRG.235496, MSTRG.262755, MSTRG.44362, MSTRG.48353, MSTRG.52182, MSTRG.52208, and MSTRG.52241. Receiver operating characteristic (ROC) curves revealed that these key lncRNAs could discriminate between cancer and cancer-adjacent tissues in patients with THCA (Fig. 6).

### Differential gene expression analysis based on the RNA-seq data of sample collected from TCGA database

**Figure 7:**
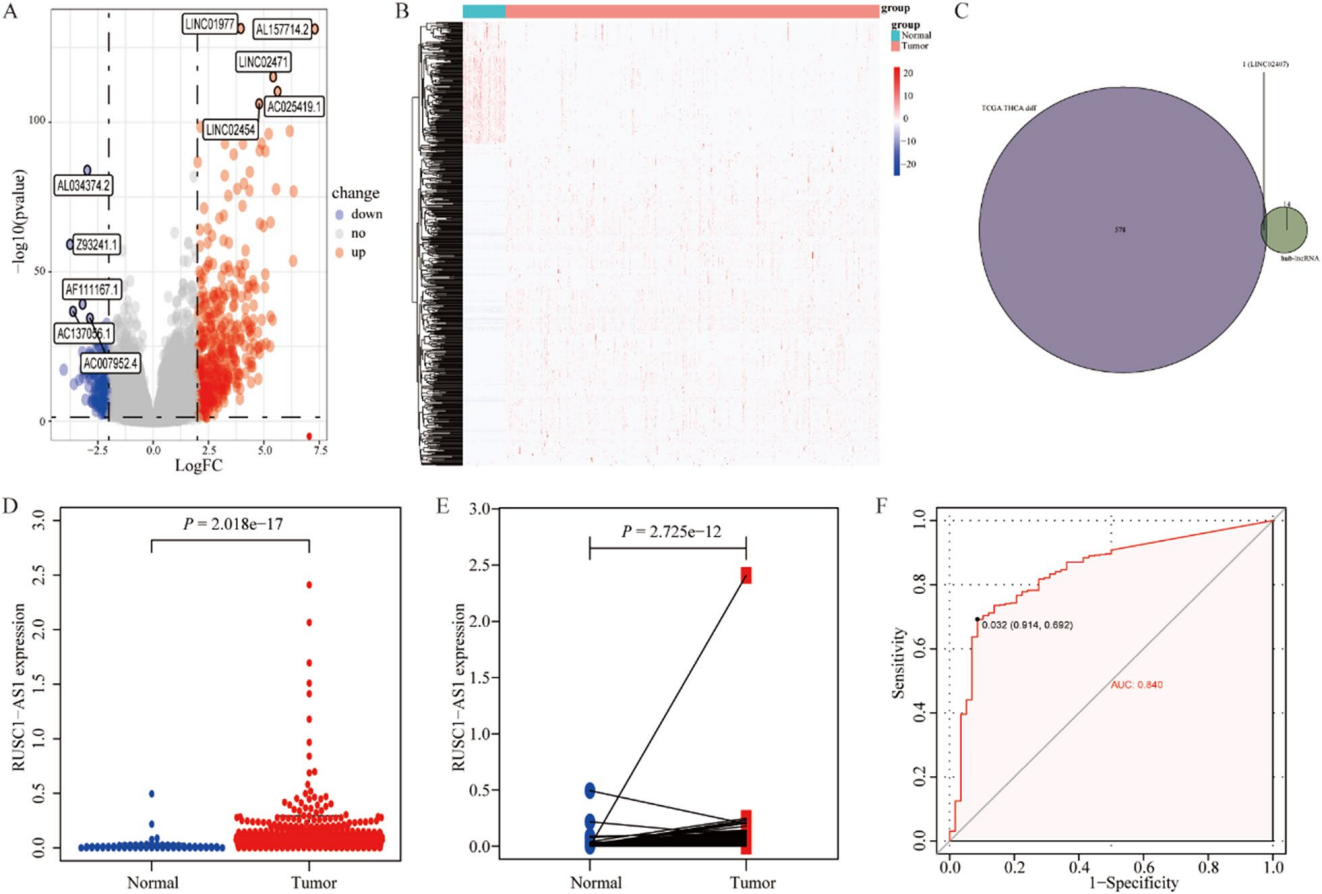
Analysis of differentially expressed lncRNA in tissues from patients with THCA in TCGA database. (**A**) Volcano plots and (**B**) heatmaps showing differentially expressed lncRNAs between THCA cancer and cancer-adjacent tissues in TCGA database. (**C**) Venn diagram showing the intersection between significantly differentially expressed lncRNAs and key lncRNAs. (**D, E**) Comparison of the levels of expression of LINC02407 (**D**) between tumor tumor-adjacent tissues and (**E**) between tumor and paired paracancerous tissues of the patients with THCA. (**F**) ROC curve analysis shows that LINC02407 enabled good discrimination between cancer and cancer-adjacent tissues of patients with THCA.

We performed differential expression analysis of lncRNAs in patients with THCA using TCGA database. We identified 579 DEGs, of which 415 were significantly upregulated and 163 were significantly downregulated. Volcano and heat maps are shown in Fig. 7A and B, respectively. Subsequently, analysis of the interaction between differentially expressed lncRNAs and key lncRNAs using a Venn diagram identified LINC02407 as the key lncRNA (Fig. 7C).

In patients with THCA in TCGA database, LINC02407 gene expression was significantly higher in tumors than in cancer-adjacent tissues (*P* < 0.001; Fig. 7D) and matched paracancerous tissues (*P* < 0.001; Fig. 7E). ROC curve analysis showed that LINC02407 expression could better distinguish between cancerous and cancer-adjacent tissues in patients with THCA (area under the curve [AUC] = 0.840; Fig. 7F).

### Correlation between the expression of the LINC02407 target gene and immune infiltration in patients with THCA

**Figure 8:**
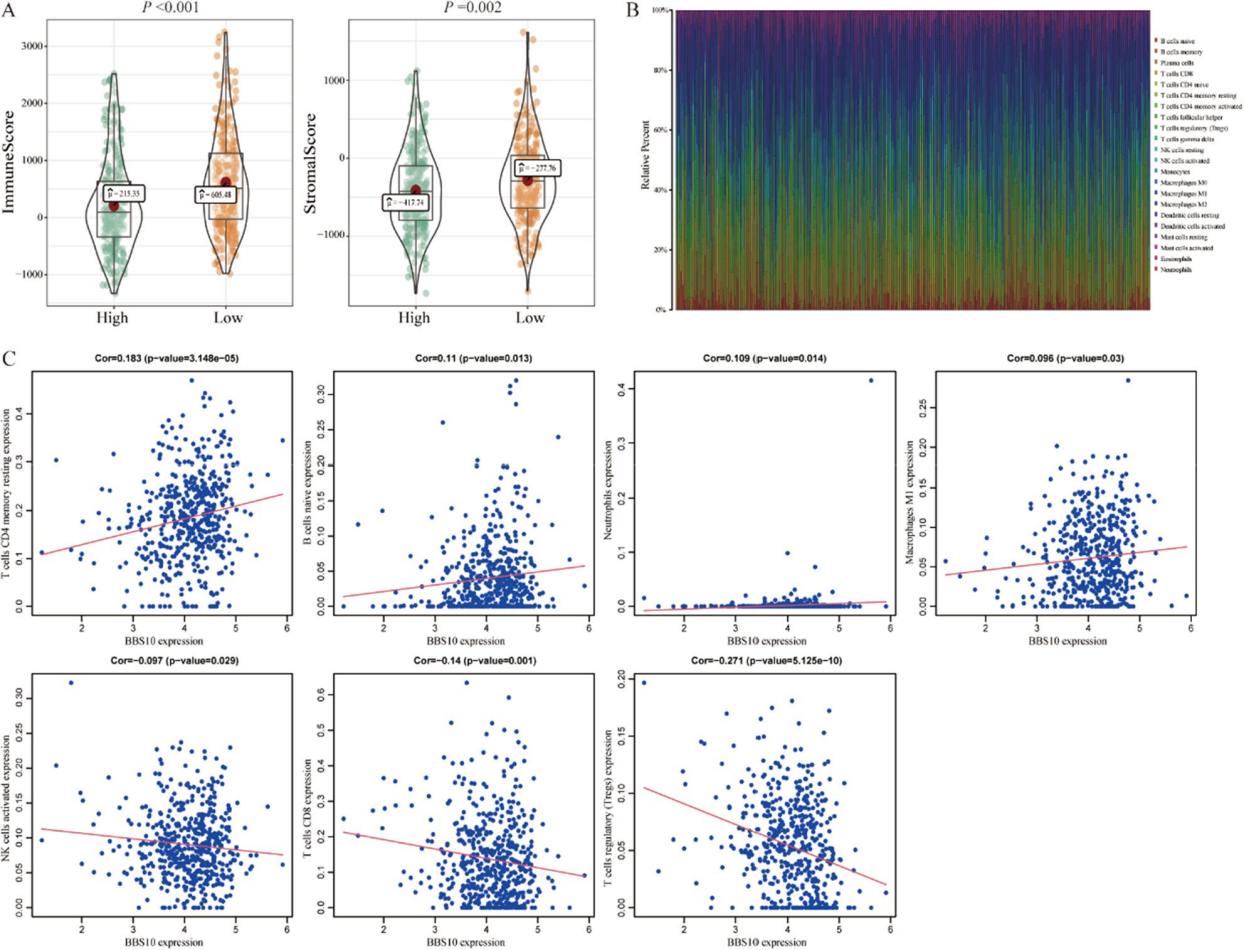
Association between BBS10, the target gene of LINC02407 expression, and infiltration of different types of immune cells. (**A**) The immune- and matrix-related scores of patients with THCA in the high-and low-expression groups. A *P*-value of < 0.05 was considered significant. (**B**) The bar graph represents the overall proportion of 22 immune cell infiltration levels in patients with THCA based on information in TCGA database. (**C**) Correlation analysis between the expression levels of various immune-cell subtypes and expression of the BBS10 gene in patients with THCA. BBS10 gene expression correlated positively with T cells CD4 memory resting, B cells naïve, Neutrophils, and Macrophages M1. In contrast, a close negative correlation was observed between BBS10 gene expression and NK cells activated, T cells CD8, and T cells regulatory (Tregs).

Using the data of patients with THCA in TCGA database, we evaluated the correlations between the expression of BBS10, the LINC02407 target gene, and the overall characteristics of 22 different immune cell subsets. The immune-related and matrix-related scores of patients in the high-BBS10-expression group were significantly lower than those in the low-BBS10-expression group (*P* < 0.001 and 0.002, respectively; Fig. 8A). We also analyzed the infiltration levels of 22 different types of immune cells in patients with THCA (Fig. 8B). The estimation of correlations between the expression of BBS10 and infiltration levels of different immune cells revealed that BBS10 expression was positively correlated with T cells CD4 memory resting, naïve B cells, neutrophils, and Macrophages M1, and negatively correlated with activated natural killer (NK) cells, activated CD8^+^ T cells, and regulatory T cells (Tregs) (Fig. 8C).

### Correlation of LINC02407 gene expression with clinical features and patient prognosis

**Figure 9:**
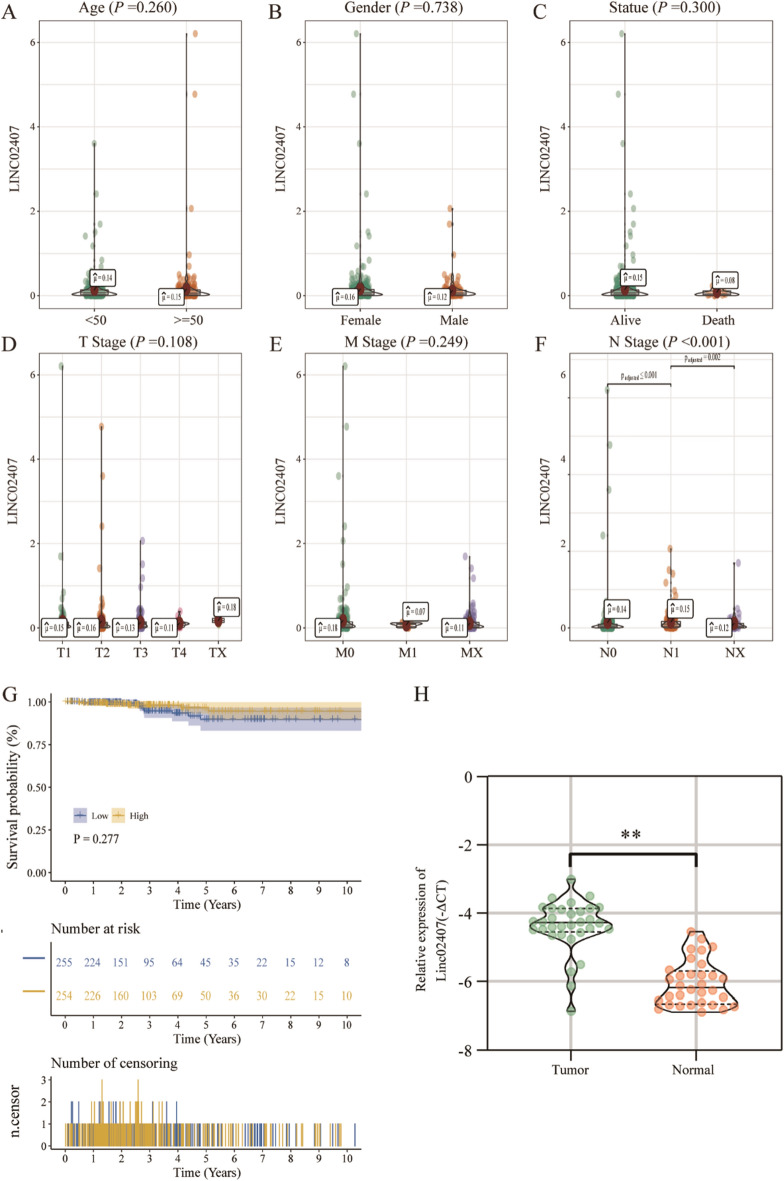
Correlation analysis between clinicopathological characteristics and LINC02407 gene expression in patients with THCA: (**A**) age, (**B**) gender, (**C**) status, (**D**) T stage, (**E**) M stage, and (**F**) N stage. LINC02407 expression and the N stage in patients were significantly correlated (*P* < 0.001), wherein the other clinical features, including age, gender, T stage, and M stage, did not show a correlation (*P* > 0.05). (**G**) Survival analysis of patients with high or low LINC02407 expression (log-rank *P* = 0.277). (**H**) Differences in the expression of LINC02407 in cancer and normal tissues (n = 32, ** *P* < 0.01).

The correlation between LINC02407 expression and the clinicopathological features of patients with THCA was assessed using Kruskal–Wallis and Wilcoxon rank-sum tests. The results showed no significant correlation was observed between LINC02407 expression and age, sex, T stage, or M stage (*P* > 0.05; Fig. 9A–E), whereas a significant negative correlation with the N stage of patients was observed (*P* < 0.001; Fig. 9F). Furthermore, we analyzed the association between LINC02407 expression and prognostic outcomes in terms of OS. Survival analysis showed no significant correlation between the survival prognosis of patients with high or low LINC02407 expression (log-rank *P* = 0.277; Fig. 9G). We conducted qPCR experiments on 32 pairs of specimens (cancer and normal tissues) in our hospital's specimen bank, and found that the expression of LINC02407 in cancer tissue was significantly higher than that in normal tissue. (*P* < 0.01; Fig. 9H).

## Discussion

PTC is the most common histological type of differentiated THCA and has a favorable prognosis. However, current diagnostic methods and treatments cannot meet all clinical needs, especially for early diagnosis and a certain recurrence rate^7^. Mounting evidence suggests that lncRNAs may be good predictors of cancer recurrence and biomarkers for diagnosing PTC at an early stage. For instance, Chen et al.^19^ demonstrated the potential of lncRNA TTTY10 as a predictive marker of PTC recurrence. Another study identified five key lncRNAs, including SLC26A4-AS1, RNF157-AS1, NR2F1-AS1, ST7-AS1, and MIR31HG that could help in diagnosing PTC^20^. In addition, lncRNA-miRNA-mRNA ceRNA networks have been reported to support disease prognosis^20^. However, the exact pathogenesis of PTC and its therapeutic targets remains unclear. In this study, we analyzed the differential expression of genes in cancer and cancer-adjacent tissues in clinical samples obtained from patients with THCA (20 pairs of PTC and cancer-adjacent tissue samples) and the GDC portal of the TGCA database (508 tumor and 58 normal tissue samples). Subsequently, using a set of bioinformatics tools such as GO, KEGG, GSEQ, PPI network, and screening for hub lncRNAs, we identified LINC02407 as a potential biomarker for diagnosing and predicting lymph node metastasis in PTC.

LINC02407 was previously shown to be associated with gastric adenocarcinoma^21^. In gastric cancer (GC) cell lines and tissue samples, LINC02407 expression was significantly upregulated, suggesting that LINC02407 plays an important role in GC progression. Furthermore, LINC02407 increases malignancy, promotes invasion of GC cells, decreases apoptosis, and controls the availability of miRNAs that can be activated by LINC02407. It has also been suggested that LINC02407 is closely related to CASC19 and cancer cell survival and affects GC via the LINC02407-miR-6845-5p/miR-4455/ADGRD1 pathway.

Here we demonstrated that LINC02407 expression in cancer tissues was significantly higher than that in adjacent tissues (*P* < 0.001) in TCGA data for patients with THCA. In addition, ROC curve analysis showed that LINC02407 expression could better distinguish between cancerous and adjacent tissues in patients with THCA (AUC = 0.840). These results indicate that measuring LINC02407 expression enables a highly sensitive and specific THCA diagnosis. We observed a close correlation between LINC02407 expression and the N stage of patients (*P* < 0.001) but no significant correlation with other clinical features, including age, sex, T stage, and M stage (*P* > 0.05), which may be related to the lower malignancy of PTC. These results suggest that LINC02407 is valuable for the early diagnosis of PCT and prediction of lymph node metastasis. However, the correlation between LINC02407 expression and the associated clinicopathological factors requires further study.

The tumor microenvironment, which also contains non-cancerous cells and tumor components (including the molecules they produce and release), is a hot topic in cancer therapy^22^. However, recent data on the immune microenvironment of thyroid tumors are often conflicting^23^; therefore, further studies are required to gather more evidence. Previous findings have suggested that neutrophils are involved in THCA growth^24^, possibly by suppressing CD8^+^ T cells or attracting metastatic cells to new sites^25,26^. In our study, we found that the expression of the LINC02407 target gene, BBS10, correlated positively with neutrophils but negatively with T cells CD8, suggesting that LINC02407 may also help regulate the tumor microenvironment of PTC.

In this study, we performed GO and KEGG analyses of differentially expressed lncRNA target genes to investigate the signaling pathways of these potential target genes in PTC. These differentially expressed lncRNA target genes mainly affected pathways such as the TGF-β signaling pathway and long-term potentiation. The TGF-β signaling pathway may mediate pro-tumor effects by regulating genomic instability, epithelial-to-mesenchymal cell-type transition, neovascularization, immune evasion, and/or metastasis^27^ in various cancers, such as cervical cancer^28^, lung cancer^29^, colorectal cancer^30^, and other types of cancer. Taken together, our results suggest that the TGF-β signaling pathway is involved in the development and lymph node metastasis of PTC, which could assist in developing a new therapeutic approach to treat PTC.

In recent years, machine learning methods for predicting the association between lncRNAs and complex human diseases have become increasingly popular among researchers. Although biological experiments and clinical methods are efficient and reliable, they are time consuming and expensive^31,32^. Computational models can provide the most promising lncRNA-disease associations for further experimental validation, reducing the time and cost of biological experiments^33^. Chen et al.^33^ established the lncRNA disease association prediction model-LRLSLDA for the first time in 2013. This model can effectively identify potential disease-lncRNA associations on a large scale and is a semi-supervised method that does not require information from negative samples. This has laid a solid theoretical foundation for lncRNA-disease association prediction research. With further research, related lncRNAs based on machine learning can be used as predictive biomarkers for the treatment and prognosis of glioblastoma, colorectal cancer, lung cancer, bladder cancer, and other tumors^31,34–36^. This also provides us with follow-up research ideas. The computational model for identifying lncRNA biomarkers of complex human diseases can be used as the future direction for biomarker identification research for papillary thyroid carcinoma.

However, this study had some limitations. First, we only used TCGA database and next-generation sequencing results from clinical specimens for bioinformatics analysis. Further experimental verification of these results at the molecular, cellular, and organismal levels is required. Second, no corresponding clinical correlation research was conducted for the clinical cases examined by next-generation sequencing, and further analysis was not performed in combination with clinical information. Due to the lack of complete clinical data in TCGA database, the sample size of the multivariate Cox analysis was relatively small, resulting in low statistical power. Third, this study does not involve machine learning processes, we will conduct further research in combination with machine learning in the future. Fourth, PTC has a good prognosis, and it was easy to find no significant difference in survival times and recurrence rates via statistical analysis. These limitations highlight the necessity of conducting a study with a larger sample size and long-term follow-up to improve statistical power and obtain more meaningful results.

## Conclusions

In conclusion, a comprehensive bioinformatics analysis of the RNA-Seq data of (i) 20 THCA patients and (ii) the THCA dataset from TCGA database identified 15 key differentially expressed lncRNAs and revealed the possible underlying molecular mechanisms and key pathways involved in PTC. Our results suggested that LINC02407 is a potential biomarker for diagnosing and predicting lymph node metastasis in PTC. However, larger prospective studies are necessary to determine the clinical value of LINC02407 and further experimental validation is required to demonstrate the biological role of LINC02407 in PTC.

## Methods

### Datasets

**Figure 10:**
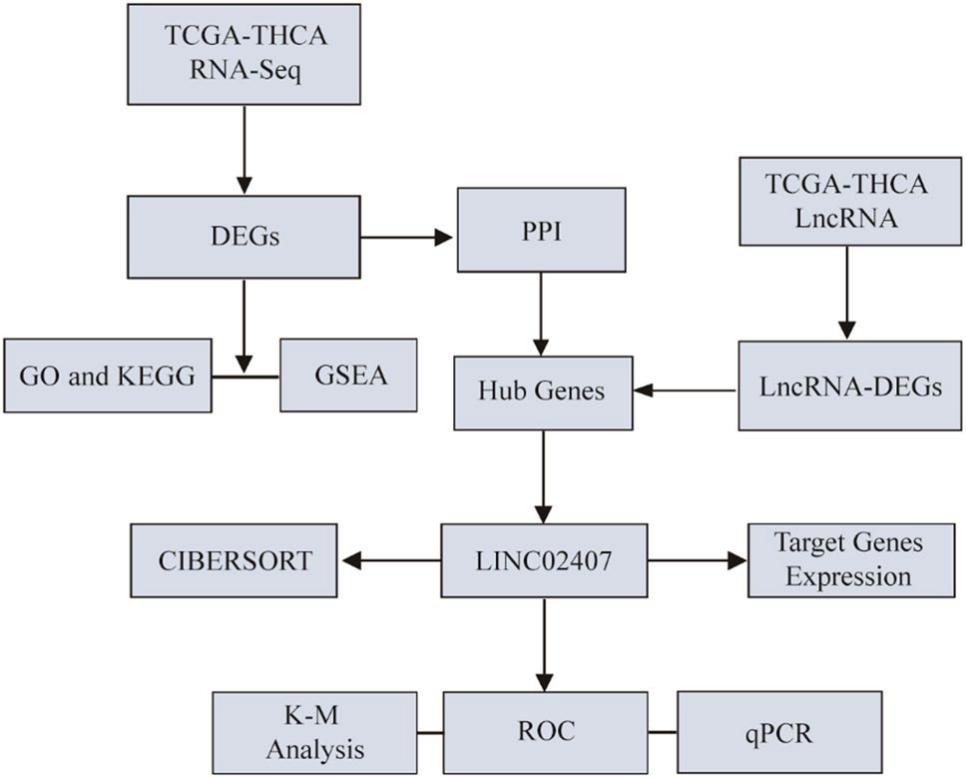
overview of the workflow.

The gene-expression data (FPKM values) in tumor (n = 510) and normal (n = 58) tissues of patients with THCA (determined by RNA sequencing [RNA-Seq]) were downloaded from the official TCGA GDC website (https://portal.gdc.cancer.gov/). We divided the expressed genes into mRNAs and lncRNAs and converted the FPKM values into transcript per million values for subsequent analysis. In addition, the clinicopathological characteristics of THCA and prognoses of the corresponding patients were downloaded from the UCSC Xena website^37^ (http://xena.ucsc.edu/); the follow-up information and clinical phase of one patient were missing in each case. After excluding patients with missing clinical data, we obtained data for 508 tumor and 58 normal tissue samples. The specific clinical information of THCA patients is shown in Table 1. The overview of the workflow is shown in Fig. 10.

**Table 1:** Baseline data of patients with THCA in TCGA database.

Variables	All patients (n = 508)	Low expression (n = 254)	High expression (n = 254)	*P*-value
Gender				0.765
Female	369 (72.6%)	183 (72.0%)	186 (73.2%)	
Male	139 (27.4%)	71 (28.0%)	68 (26.8%)	
Age				0.372
< 50	282 (55.5%)	136 (53.5%)	146 (57.5%)	
≥ 50	226 (44.5%)	118 (46.5%)	108 (42.5%)	
T				0.018
T1-2	308 (60.6%)	167 (65.7%)	141 (55.5%)	
T3-4&TX	200 (39.4%)	87 (34.3%)	113 (44.5%)	
N				< 0.001
N0	228 (44.9%)	138 (54.3%)	90 (35.4%)	
N1&NX	280 (55.1%)	116 (45.7%)	164 (64.6%)	
M				0.152
M0	286 (56.3%)	135 (53.1%)	151 (59.4%)	
M1&MX	222 (43.7%)	119 (46.9%)	103 (40.6%)	

A *P*-value of < 0.05 was considered significant.

### DEGs

To analyze the significant DEGs between cancer and cancer-adjacent tissues of patients with THCA, we analyzed 20 pairs of cancer and cancer-adjacent tissues using RNA-seq. All specimens were obtained from patients who underwent standard surgical procedures between January 2016 and December 2018 at the Department of Head and Neck Surgery, Renji Hospital, School of Medicine, Shanghai Jiaotong University. The specimens were stored in liquid nitrogen at − 80 °C immediately after removal. The patients did not receive radiotherapy or chemotherapy before surgery. Pathological examination confirmed PTC. This study was approved by the Ethics Committee of Renji Hospital, School of Medicine, Shanghai Jiaotong University (Shanghai, China). All participants provided written informed consent before enrollment. All methods were performed according to the relevant guidelines and regulations. Differences between gene expression in cancer and cancer-adjacent tissues were shown by a PCA plot using the FactoMineR package of the R software^38^. We used the DEseq2 package^39^ for differential expression analysis, and genes satisfying the screening criteria [| log_2_ FC|≥ 1 and q < 0.05] were identified as significant DEGs.

Subsequently, we counted the DEGs between cancer and cancer-adjacent tissues of patients with THCA using the information deposited in TCGA database. DEGs between cancer and cancer-adjacent tissues were analyzed using DEseq2^39^. The thresholds for differential gene expression were |log_2_ FC|> 2 and an adjusted *p*-value < 0.05. The results of the differential gene expression analyses were analyzed by generating heat maps and volcano plots.

### Functional enrichment analysis and GSEA

GO analysis is a common method for conducting large-scale functional-enrichment studies and determining the associated BPs, MFs, and CCs. The KEGG database is a widely used database that stores information on genomes, biological pathways, diseases, and drugs. In this study, we used the clusterProfiler R software package^40^ to perform GO annotation and KEGG pathway enrichment analysis of the signature genes. The cutoff value of a false-discovery rate of < 0.05 was considered the threshold for a statistically significant difference.

To investigate differences in BPs between different subgroups, we performed GSEA based on the gene expression profiling dataset of patients with THCA. GSEA is a computational method used to analyze whether a particular gene set is significantly different between two biological states, and is often used to estimate changes in the pathway and BP activities in samples between different datasets^41^. We downloaded the “c2.all.v7.0.entrez.gmt” gene set from the MSigDB database^42^ (https://www.gsea-msigdb.org/gsea/msigdb) for GSEA, and the differences with an adjusted *P*-value of < 0.05 were considered statistically significant.

### Comparison of the immune cell infiltration levels and immune-related scores between two groups

To quantify the proportions of different immune cells in THCA samples, we used the CIBERSORT algorithm and LM22 gene set to analyze 22 human immune cell phenotypes (including B cells, T cells, NK cells, and macrophages) in the tumor immune microenvironment for a highly sensitive and specific distinction^43^. CIBERSORT is a deconvolution algorithm that uses a set of reference gene expression values (547 eigengenes). A group of genes is considered the smallest representative of each cell type; the values in these groups were then used to infer data for diverse cell-type proportions from bulk-tumor sample data. Pearson’s correlation coefficient was used to calculate the relationship between the infiltration levels of different immune cells and the expression levels of key lncRNA target genes.

The ESTIMATE algorithm was used to quantify the immune activity (level of immune infiltration) in a tumor sample based on gene expression profiles. We assessed the immune activity of each tumor sample and its stromal score using the ESTIMATE package in R^44^. The Mann–Whitney U test was used to compare the levels of infiltrating immune cells between the two groups of samples.

### Clinical prognosis-correlation analysis

We evaluated the impact of the expression of key lncRNAs on the clinicopathological characteristics of the patients. Subsequently, by combining the expression of key lncRNAs with clinicopathological characteristics, their independent predictive power for OS was analyzed using univariate and multivariate Cox regression models.

### Construction of a ceRNA network

For the differentially expressed lncRNAs obtained by RNA-Seq, we performed cis- and trans-target analyses to predict possible mRNA-target information through co-expression analysis. Before performing basic statistical analysis, we downloaded information for miRNA–mRNA interactions from the miRTarBase database . Subsequently, we predicted potentially regulated miRNAs from the mRNA information based on the miRTarBase database. Cytoscape (v3.7.2)^45^ was used to construct a ceRNA network.

### Construction of a PPI network and screening for hub genes

In this study, we used online STRING^46^ to predict PPIs and construct PPI networks for selected genes. Using the STRING database, genes with scores > 0.4 were selected to build the network model, which was visualized using Cytoscape (v3.7.2)^45^. The MCODE plugin was used to localize high-density regions in the map based on the vertex-weighting scheme, and high-density regions were treated as hub genes.

### Statistical analysis

R software (version 4.0.2) was used to process and analyze the data generated in this study. To compare two groups of continuous variables, we used the independent Student’s t-test for normally distributed variables. Differences among non-normally distributed variables were analyzed using the Mann–Whitney U test (Wilcoxon rank-sum test). Chi-square or Fisher’s exact tests were used to compare and analyze the statistical significance between the two groups of categorical variables. Correlation coefficients between different genes were calculated using Pearson’s correlation analysis. The survival package in R was used for survival analysis, the Kaplan–Meier survival curve was used to show survival differences, and the log-rank test was used to evaluate the significance of survival time differences between the two groups of patients. Univariate and multivariate Cox analyses were used to identify independent prognostic factors. ROC curves were drawn using the pROC package of the R software^47^, and the AUC was calculated to assess the accuracy of the ROC curves in distinguishing cancer from cancer-adjacent tissues. All statistical *P* values were two-sided, and *P* < 0.05 was considered to indicate a statistically significant difference.

### Ethics approval and consent to participate

This study was approved by the Ethical Committee of Renji Hospital, School of Medicine, Shanghai Jiao Tong University (2018–159), and all participants provided informed consent.

## Data Availability

Publicly available datasets were analyzed in this study. This data can be found here: https://portal.gdc.cancer.gov/.
